# A randomized controlled study to assess the effect of mosapride citrate on intestinal recovery following gastrectomy

**DOI:** 10.1038/s41598-024-57870-w

**Published:** 2024-03-25

**Authors:** Shiyeol Jun, Seyeol Oh, Ji Eun Jung, In Gyu Kwon, Sung Hoon Noh

**Affiliations:** Department of Surgery, Gangnam Severance Hospital, Yonsei University College of Medicine, Seoul, Korea

**Keywords:** Gastric cancer, Surgical oncology

## Abstract

The enhanced recovery after surgery (ERAS) protocol, including prokinetic medications, is commonly used to prevent postoperative ileus. Prospective studies evaluating the effectiveness of mosapride citrate, a prokinetic 5-hydroxytryptamine 4 receptor agonist, in patients undergoing gastrectomy within the ERAS framework are lacking. This double-blind randomized trial included patients who were scheduled for laparoscopic or robotic gastrectomy for gastric cancer. Participants were randomly assigned to either a control (placebo) or experimental (mosapride citrate) group, with drugs administered on postoperative days 1–5. Bowel motility was evaluated based on bowel transit time measured using radiopaque markers, first-flatus time, and amount of food intake. No significant differences were observed in baseline characteristics between the two groups. On postoperative day 3, no significant difference was observed in the number of radiopaque markers visible in the colon between the groups. All factors associated with bowel recovery, including the time of first flatus, length of hospital stay, amount of food intake, and severity of abdominal discomfort, were similar between the two groups. Mosapride citrate does not benefit the recovery of intestinal motility after minimally invasive gastrectomy in patients with gastric cancer. Therefore, routine postoperative use of mosapride citrate is not recommended in such patients.

## Introduction

The enhanced recovery after surgery (ERAS) protocol is a multidisciplinary approach that aims to accelerate postoperative recovery, shorten hospital stays, and reduce healthcare costs^1^. Prevention of postoperative ileus following abdominal surgery is an important aspect of the ERAS protocol^2^.

Postoperative ileus is primarily caused by the inhibition of sympathetic neural reflexes due to anesthesia and inflammatory responses resulting from surgical manipulation^3–5^, and can delay recovery and increase the risk of other complications, including atelectasis and nosocomial infections^6^.

Mosapride citrate is a widely used prokinetic medication that is administered as both preventive and curative measures for postoperative ileus^7,8^. It stimulates 5-hydroxytryptamine 4 (5-HT_4_) receptors located on neurons of the gastrointestinal tract, thereby promoting bowel motility^9^. Additionally, it acts on α7 nicotinic acetylcholine receptors and consequently suppresses the inflammatory response of macrophages, which is another major pathogenic mechanism of ileus development^10^.

A recent study reported that mosapride significantly reduced flatus and defecation time in patients who underwent laparoscopic colectomy^11^. However, prospective studies on the effectiveness of mosapride in patients undergoing minimally invasive gastrectomy are lacking.

Hence, this study aimed to assess the efficacy of mosapride in patients undergoing gastrectomy. Specifically, we aimed to determine whether mosapride offered additional advantages in patients undergoing minimally invasive surgery performed in accordance with ERAS protocols^12,13^.

## Methods

### Study design

This double-blind, placebo-controlled randomized trial was conducted at Yonsei University Gangnam Severance Hospital, Seoul, South Korea. The study was conducted in compliance with the Declaration of Helsinki and approved by the Gangnam Severance Institutional Review Board. The trial is registered on ClinicalTrials.gov (NCT04493125, 28/07/2020), and written informed consent was obtained from all participants.

### Patient selection

Patients aged 20–80 years with pathologically confirmed gastric adenocarcinoma scheduled for radical gastrectomy with lymph node dissection via laparoscopic or robotic approaches were eligible for inclusion^14^. Exclusion criteria comprised the presence of other malignancies, distant metastasis, an American Society of Anesthesiologists physical status score of 4 or 5, and conditions affecting intestinal motility such as prior bowel obstruction, major abdominal surgery, or uncontrolled diabetes mellitus^15,16^. Dropout criteria included patients undergoing additional bowel resection, extensive adhesiolysis, or conversion to laparotomy during surgery^6,8^. Additionally, patients allergic to mosapride or unable to consume oral medication were also designated for dropout.

### Treatment and assessments

Participants were randomly assigned to either the control or experimental group by an independent clinical research coordinator not involved in surgery or postoperative care. Both patients and treating physicians remained unaware of the group assignment. The experimental group received 5.29 mg of mosapride thrice daily on postoperative days 1–5, whereas those in the control group received placebo pills (Placebo Pharmaceutical Co., Otsu city, Japan) following the same schedule.

**Figure 1:**
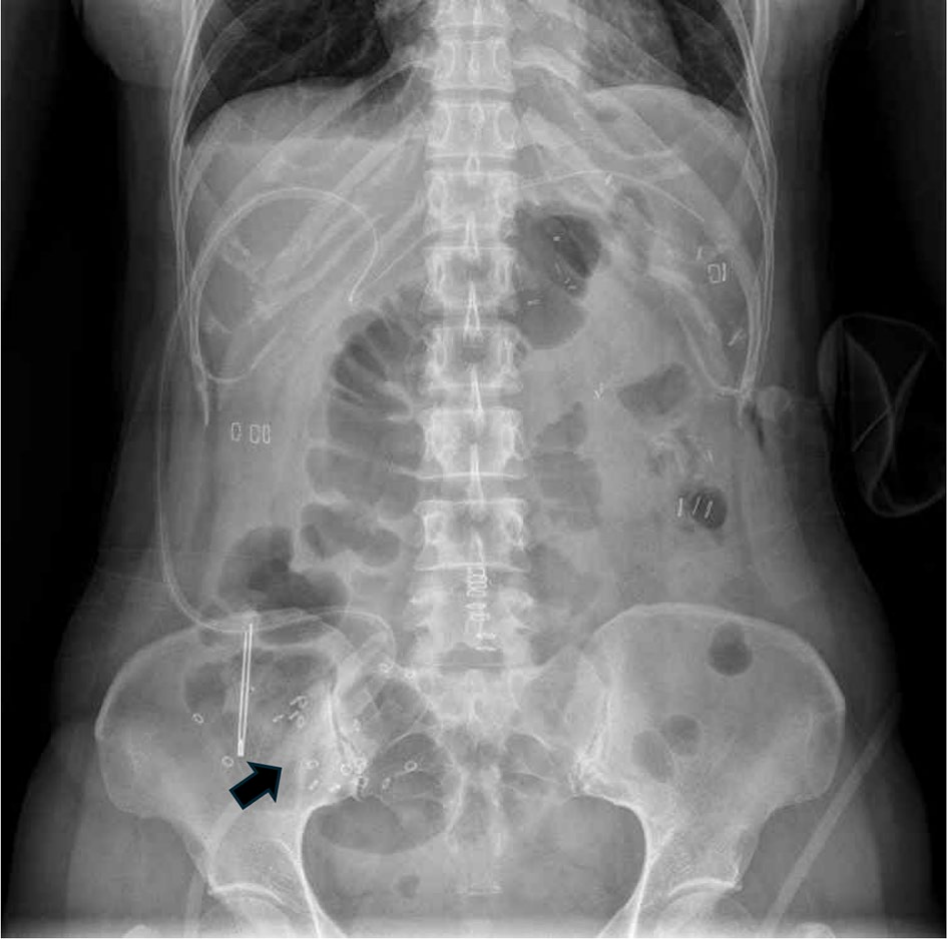
Representative radiographic image of radiopaque markers observed in the colon. (Black arrow).

During surgery, radiopaque markers within a capsule were placed at the bowel anastomosis site. Bowel transit time was measured by counting the visible radiopaque markers on plain radiographic images of the stomach, small bowel, or colon taken on postoperative days 1, 3, and 5 (Fig. 1)^17^. Laboratory examinations, including white blood cell (WBC) count, neutrophil count, and C-reactive protein levels, were conducted on the same day to assess inflammatory responses. Postoperative complications were classified according to the Clavien–Dindo classification^18^.

All patients adhered to the ERAS guidelines, encompassing anesthetic management, near-zero fluid balance, minimal opioid usage, avoidance of nasogastric tube insertion, early enteral nutrition, and prompt mobilization^12,13^. Epidural anesthesia was not administered during the study, and any additional analgesic administrations were diligently documented.

Patients followed a standardized diet protocol, including water sips on postoperative day 1, a liquid diet on postoperative day 2, and a soft diet on postoperative day 3, with possible delays based on individual tolerance levels. Food intake was assessed by evaluating the proportion of meals consumed. Daily abdominal discomfort was assessed until postoperative day 5 using a questionnaire based on the numerical rating scale, with scores ranging from 1 (very comfortable) to 5 (very uncomfortable).

The primary endpoint was the bowel transit time, assessed by counting radiopaque markers in the colon on postoperative day 3. Secondary endpoints included food intake amount, severity of abdominal discomfort, time to first flatus/defecation, and levels of inflammatory markers.

### Statistical analysis

With a 10% dropout rate and 1:1 randomization, we aimed for 80% power with a two-sided significance level of 5%. On prior research, mosapride brought 1.4 times improvement in first defacation time. Therefore, we hypothesized at least a 1.3-fold improvement of bowel movement, assessed by the number of radiopaque markers passing into the colon in the experimental group compared to the control group^11^. Thus, a minimum of 52 patients per group was required.

Categorical variables are presented as numbers and proportions, and continuous variables as mean ± standard deviation. Categorical variables were analyzed using the chi-squared or Fisher’s exact test, whereas the Kruskal–Wallis test was employed for ordinal variables. Continuous variables were assessed using Student’s t-test. Repeated-measures ANOVA was used to comprehensively compare the differences between the two groups over time. Statistical significance was set at *P* < 0.05. All statistical analyses were conducted using SPSS software version 22 (IBM Corp., Armonk, NY, USA).

## Results

### Patient characteristics

**Figure 2:**
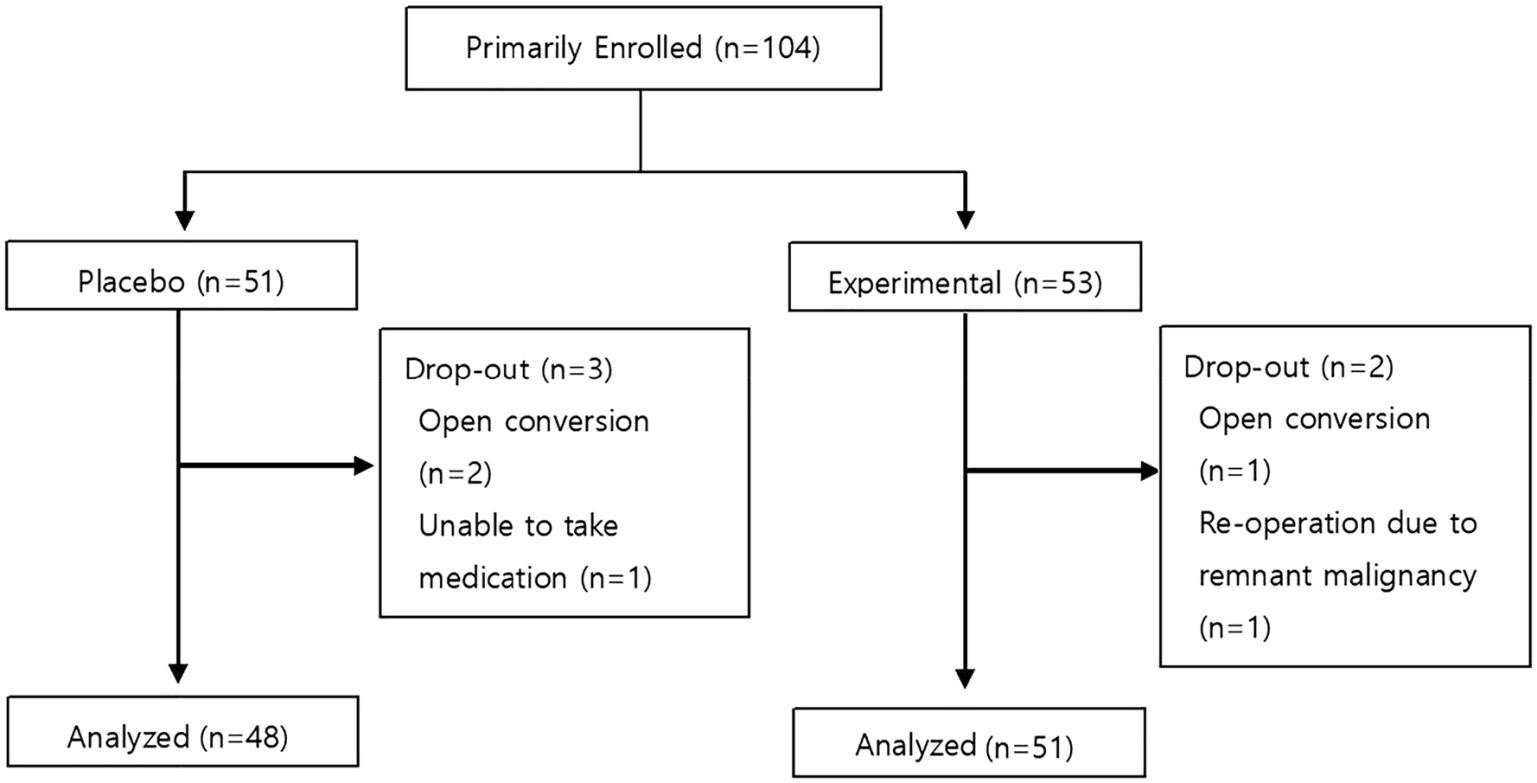
Flow chart of patient selection.

In total, planned 104 patients were enrolled between July 2020 and March 2021. Among them, 51 and 53 patients were allocated to the experimental and control groups, respectively. Three patients in the control group and two in the experimental group dropped out (Fig. 2).

Baseline characteristics such as age, medical history, type of operation, blood loss during the surgery, and operation time were well balanced between the two groups. However, the experimental group had a slightly higher proportion of women compared with the control group, although this difference was not statistically significant (54.9% vs. 35.4%; *P* = 0.052; Table 1).

**Table 1:** Patient characteristics.

Characteristic	Control group (n = 48)	Experimental group (n = 51)	*P*-value
Age (years)	60.02 ± 10.64	60.59 ± 10.34	0.789
Sex			
Male	31 (64.6%)	23 (45.1%)	
Female	17 (35.4%)	28 (54.9%)	0.052
ASA			0.758
I	9 (18.8%)	8 (15.7%)	
II	29 (60.4%)	36 (70.6%)	
III	10 (20.8%)	7 (13.7%)	
BMI (kg/m^2^)	24.67 ± 3.58	24.23 ± 2.90	0.758
Blood loss (cc)	71.88 ± 78.92	57.28 ± 93.37	0.733
Operation time (min)	212.65 ± 35.16	206.07 ± 38.98	0.449
Approach method			0.135
Laparoscopic	45 (93.8%)	43 (84.3%)	
Robotic	3 (6.3%)	8 (15.7%)	
Type of resection			0.249
Subtotal	36 (75.0%)	43 (84.3%)	
Total	12 (25.0%)	8 (15.7%)	
Anastomosis type			0.519
B-I	20 (41.7%)	21 (41.2%)	
B-II	14 (29.2%)	17 (33.3%)	
STG R-Y	2 (4.2%)	5 (9.8%)	
TG R-Y	12 (25.0%)	8 (15.7%)	

*ASA* American Society of Anesthesiologists physical status score, *BMI* Body mass index, *B-I* Billroth I, *B-II* Billroth II, *STG R-Y* Subtotal gastrectomy Roux-en Y, *TG R-Y* Total gastrectomy Roux-en Y.

### Motility evaluation

No hazardous surgical events occurred during the insertion of radio-opaque markers. However, radio-opaque marker insertion was missed in seven patients during surgery: three patients in the placebo group and four patients in the experimental group. The analysis of the number of radiopaque markers was conducted only on patients who did not miss radiopaque marker insertion in each group, 45 patients in the control group, 47 patients in the experimental group.

On postoperative day 3, the average numbers of radiopaque markers detected in the colon were 14.0 ± 7.71 and 13.30 ± 7.50 in the control and experimental groups, respectively. We observed no statistically significant differences between the groups (*P* = 0.659; Table 2).

**Table 2:** Clinical outcome associated with the gastrointestinal motility.

	Control group (n = 48)	Experimental group (n = 51)	*P-*value
Number of markers in small bowel (n)			
POD1	12.94 ± 8.62	11.76 ± 8.87	0.506
POD3	3.60 ± 5.88	4.28 ± 6.05	0.588
POD5	0.43 ± 1.59	0.87 ± 2.79	0.361
Number of markers in colon (n)			
POD1	0.42 ± 2.08	0.30 ± 2.04	0.780
POD3	14.00 ± 7.71	13.30 ± 7.50	0.659
POD5	17.89 ± 5.69	18.91 ± 3.28	0.290
Abdominal discomfort (NRS 1–5)			
POD1	2.46 ± 1.33	2.63 ± 1.22	0.256
POD2	2.62 ± 0.98	2.65 ± 1.01	0.897
POD3	2.71 ± 1.11	2.48 ± 0.98	0.239
POD4	2.18 ± 0.96	2.23 ± 0.93	0.828
POD5	1.84 ± 1.04	1.77 ± 0.89	0.837
Proportion of food intake (%)			
POD2	64.79 ± 21.88	65.00 ± 26.94	0.967
POD3	63.92 ± 20.97	62.43 ± 21.50	0.729
POD4	62.48 ± 22.10	64.47 ± 20.14	0.640
POD5	63.09 ± 24.35	66.57 ± 21.97	0.458
Time to first flatus (day)	3.33 ± 0.88	3.31 ± 0.79	0.907
Time to first flatus (hour)	72.44 ± 17.56	72.25 ± 19.80	0.457
Time to first defecation (day)	4.04 ± 0.87	4.00 ± 1.52	0.910

*POD* postoperative day.

On postoperative day 5, the average numbers of markers detected in the colon were 17.89 ± 5.69 and 18.91 ± 3.28 in the control and experimental groups, respectively; however, this difference was not statistically significant (*P* = 0.29). After conducting subgroup analysis using stratification based on the anastomosis type, no statistically significant difference was observed (Table 3).

**Table 3:** Number of radiopaque markers in colon according to anastomosis type.

	Control group	Experimental group	*P*-value
STG B-I	(n = 20)	(n = 19)	
POD1	0.75 ± 2.92	0.74 ± 3.21	0.989
POD3	14.30 ± 7.70	14.16 ± 6.40	0.950
POD5	19.00 ± 4.03	19.11 ± 2.81	0.925
STG B-II	(n = 13)	(n = 17)	
POD1	0	0	
POD3	11.08 ± 8.36	11.47 ± 8.67	0.901
POD5	14.69 ± 8.64	18.00 ± 4.51	0.185
STG R-Y	(n = 2)	(n = 3)	
POD1	0	0	
POD3	7.00 ± 9.90	10.00 ± 8.72	0.761
POD5	17.00 ± 4.24	20.00 ± 0	0.500
TG R-Y	(n = 10)	(n = 8)	
POD1	0.30 ± 0.95	0	0.343
POD3	18.60 ± 1.19	16.38 ± 6.76	0.424
POD5	20.00 ± 0	20.00 ± 0	

*POD* postoperative day, *B-I* Billroth I, *B-II* Billroth II, *STG R-Y* Subtotal gastrectomy Roux-en Y, *TG R-Y* Total gastrectomy Roux-en Y.

The levels of abdominal discomfort reported by the patients were similar in both groups. The average numeric rating scores of the questionnaire were 1.84 ± 1.04 and 1.77 ± 0.89 in the control and experimental groups, respectively (*P* = 0.837).

We observed no significant difference in the proportion of food intake between the two groups. On postoperative day 5, the average proportions of food intake were 63.09% ± 24.35% and 66.57% ± 21.97% in the control and experimental groups, respectively (*P* = 0.458). The times to the first flatus and defecation were similar between the two groups.

**Figure 3:**
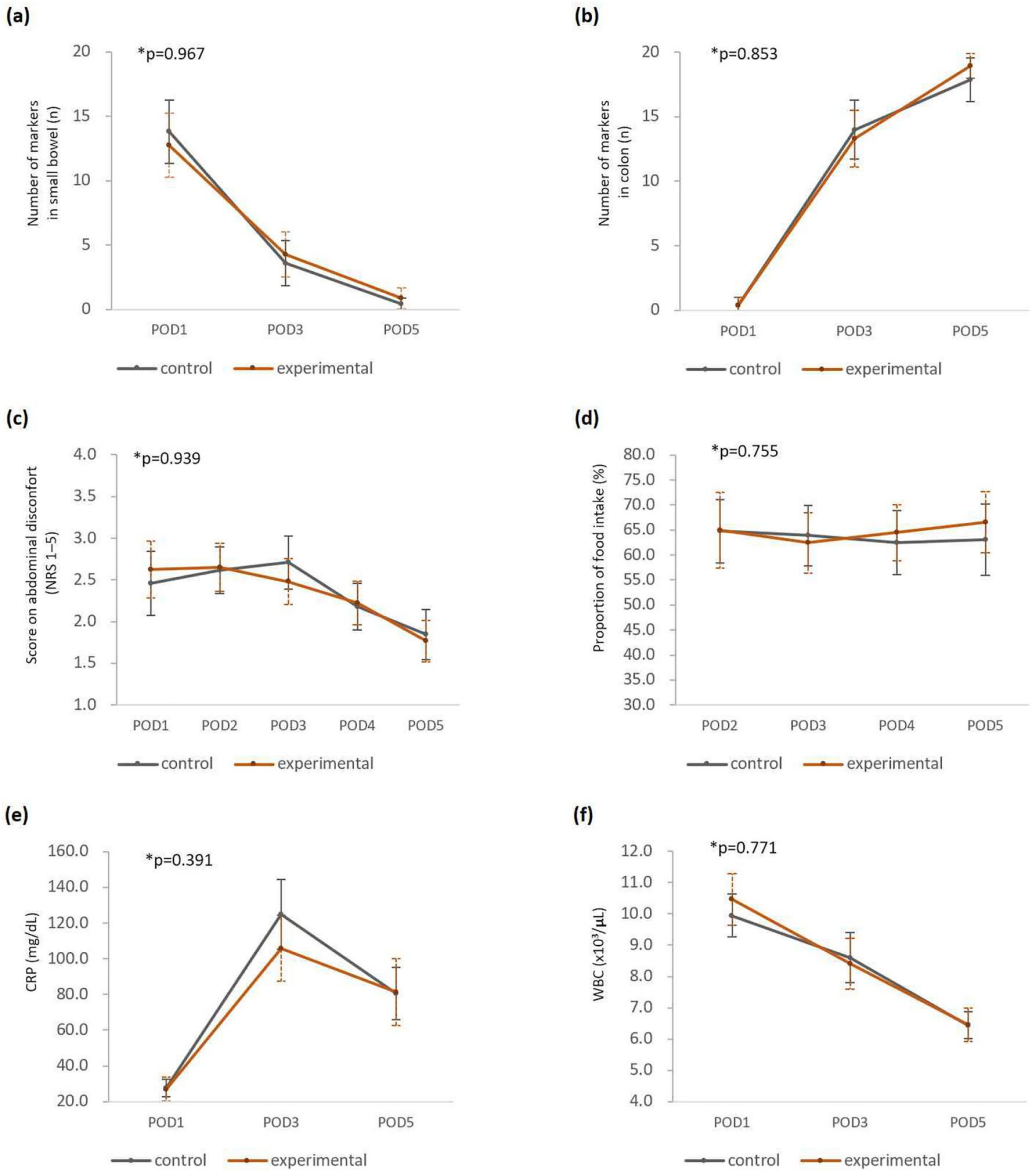
Clinical outcomes of two groups over time. Number of markers in (**a**) small bowel and (**b**) colon. (**c**) Abdominal discomfort score in numerical rating scale. (**d**) Proportion of food intake. Level of (**e**) CRP and (**f**) WBC count. *P-values compared the difference between two groups with RM-ANOVA. *POD* postoperative day; *NRS* numerical rating scale; *CRP* C-reactive Protein; *WBC* white blood cell.

When analyzing the difference between the two groups over time using RM-ANOVA, the *P*-value did not indicate a significant difference on all variables associated with bowel motility (Fig. 3).

### Postoperative outcomes

We observed no significant differences in the rate or severity of postoperative complications between the two groups. The length of hospital stays in both groups were similar (Table 4). No complications related to placebo or mosapride treatment, including allergic reactions or impaired liver function, were reported.

**Table 4:** Laboratory Findings and Postoperative Course.

	Control group (n = 48)	Experimental group (n = 51)	*P-*value
CRP level (mg/dL)			
POD1	27.68 ± 16.89	26.91 ± 24.18	0.857
POD3	124.90 ± 67.29	105.88 ± 66.00	0.159
POD5	80.48 ± 50.84	81.32 ± 66.35	0.942
WBC count (× 10^3^/µL)			
POD1	9.95 ± 2.39	10.46 ± 2.96	0.340
POD3	8.60 ± 2.78	8.41 ± 2.88	0.741
POD5	6.44 ± 1.47	6.45 ± 1.90	0.987
Neutrophil (%)			
POD1	79.63 ± 6.08	80.94 ± 5.57	0.267
POD3	73.87 ± 6.43	73.78 ± 7.55	0.951
POD5	69.09 ± 5.26	68.22 ± 7.52	0.508
Postoperative opioid injection (n)	2.02 ± 2.20	2.32 ± 2.15	0.449
Complication cases (n)	30 (62.5%)	32 (62.7%)	0.980
Grade (Clavian–Dindo)			0.994
I	13 (27.1%)	14 (27.5%)	
II	17 (35.4%)	18 (35.3%)	
Hospital stay Day	6.13 ± 1.06	5.84 ± 1.07	0.191

*POD* postoperative day, *CRP* C-reactive Protein, *WBC* white blood cell.

Of the 101 enrolled patients, 27 experienced grade I complications, and 36 experienced grade II complications. Two patients were excluded owing to intolerance to oral medications. In the experimental group, one patient required reoperation owing to obstructive symptoms and the presence of malignant cells at the surgical margin. In the control group, one patient dropped out because of delayed gastric emptying. None of the patients experienced severe complications, such as anastomotic leakage or sepsis.

Finally, we observed no significant differences in the results of the laboratory examinations for assessing inflammation between the two groups (Table 4).

## Discussion

In our study, we observed that mosapride citrate did not promote bowel motility in patients who had undergone minimally invasive gastrectomy performed in accordance with the ERAS protocol.

The ERAS protocol for patients undergoing gastrectomy includes the use of a laparoscopic approach, early resumption of postoperative oral nutrition, and minimal opioid use. However, motility-enhancing medication is only weakly recommended in the ERAS protocol for gastrectomy and its use has a very low evidence level^2^. Many prokinetic medications are associated with the 5-HT receptors. Mosapride citrate is a 5-HT_4_ receptor agonist that increases the release of acetylcholine from excitatory neurons in the stomach and duodenum^19^. However, its propulsive effect on the small intestine and colon has remained unclear in previous studies^7,20^.

Patients who undergo laparoscopic colectomy can benefit from mosapride citrate, such as experiencing a shorter defecation period, reduced incidence of ileus, and shorter hospital stays^11,21,22^. In contrast, we did not observe a positive effect of mosapride on the postoperative recovery of bowel movements. The reasons for this negative observation may be as follows. First, unlike patients who have undergone colectomy, those who have undergone distal or total gastrectomy do not retain the gastric antrum. As the gastric antrum is the main target of mosapride, patients without the gastric antrum may experience reduced effects of mosapride. We anticipate that mosapride administration in patients undergoing pylorus-preserving gastrectomy or proximal gastrectomy, in which the gastric antrum is not removed during surgery, could lead to more significant effects. Second, mosapride is not only a 5-HT_4_ receptor agonist but also a 5-HT_3_ receptor antagonist^23^. Antagonists of 5-HT_3_ receptors, such as ondansetron and alosetron, are used as antiemetic or antidiarrheal drugs. Ondansetron also slows colonic transit and inhibits the colonic motor response to meals^24,25^. Therefore, an antagonistic effect on 5-HT_3_ receptors may be the pharmacological reason for the limited promotion of colonic motility by mosapride in the enrolled patients. Lastly, surgery inherently exerts a more direct and powerful influence on the bowel, leading to delayed and subdued effect of the drug in the context of surgery compared to that of medical conditions. In medical conditions such as diabetes or Parkinson’s disease, mosapride effectively addresses constipation and decreased bowel motility. Patients with these conditions often exhibit an initial 5-HT system hypofunction in the gastrointestinal system. When administered, mosapride delivers an immediate and more pronounced impact by replacing 5-HT^26,27^. However, physical manipulation or thermal injury during surgery can affect the myenteric plexus of the gastrointestinal tract. The recovery and regeneration of neurons generally take approximately 10–14 days, as long as up to 6 weeks^28–30^. Postoperatively, 5 days may not be sufficient for neuronal recovery, leading to an unnoticed effect of the medication.

Despite the lack of positive outcomes, to our knowledge, this is the first randomized, placebo-controlled study conducted to assess the efficacy of mosapride in patients undergoing minimally invasive gastrectomy. Additionally, the randomized, prospective trial design minimizes bias. However, the fact that it was conducted at a single center and the population was limited to Asians are limitations of this study.

Postoperative recovery of bowel motility is achieved starting from the small intestine followed by the stomach to the colon^31^. The recovery of colon motility may play a crucial role in reducing abdominal discomfort and promoting gas passage. Therefore, we assumed that prokinetic medications, which mainly act on the colon, may be more effective than mosapride, which acts on the stomach and duodenum. Further research on such agents might be required to identify intestinal recovery following gastrectomy.

In conclusion, mosapride citrate does not improve the recovery of intestinal motility following minimally invasive gastrectomy in patients with gastric cancer. Therefore, the routine postoperative use of mosapride citrate is not recommended in patients who have undergone minimally invasive gastrectomy.

## Data Availability

Datasets used and/or analyzed are available from the corresponding author on reasonable request.
